# Electrospray mode discrimination with current signal using deep convolutional neural network and class activation map

**DOI:** 10.1038/s41598-022-20352-y

**Published:** 2022-09-29

**Authors:** Man Jin Kim, Jin Yeong Song, Seok Hyeon Hwang, Dong Yong Park, Sang Min Park

**Affiliations:** 1 School of Mechanical Engineering, Pusan National University, 2, Busandaehak-ro 63 beon-gil, Geumjeong-gu, Busan, 46241 Republic of Korea; 2 Smart Manufacturing Technology R&D Group, Korea Institute of Industrial Technology, 320 Techno sunhwan-ro, Yuga-eup, Dalseong-gun, Daegu, 42994 Republic of Korea

**Keywords:** Mechanical engineering, Techniques and instrumentation

## Abstract

The electrospray process has been extensively applied in various fields, including energy, display, sensor, and biomedical engineering owing to its ability to generate of functional micro/nanoparticles. Although the mode of the electrospray process has a significant impact on the quality of micro/nano particles, observing and discriminating the mode of electrospray during the process has not received adequate attention. This study develops a simple automated method to discriminate the mode of the electrospray process based on the current signal using a deep convolutional neural network (CNN) and class activation map (CAM). The solution flow rate and applied voltage are selected as experimental variables, and the electrospray process is classified into three modes: dripping, pulsating, and cone-jet. The current signal through the collector is measured to detect the deposition of electrospray droplets on the collector. The 1D CNN model is trained using frequency data converted from the current data. The model exhibits excellent performance with an accuracy of 96.30%. Adoption of the CAM configuration enables the model to provide a discriminative cue for each mode and elucidate the decision-making process of the CNN model.

## Introduction

Electrospray process is a process of spraying a solution using the electrostatic force induced by high voltage to produce fine droplets. Nollet first observed the electrohydrodynamic atomization phenomenon in the seventeenth century after which Rayleigh defined the limit of charge in a droplet^1–3^. The formation of fine droplets in electrospraying is dependent on the hydrostatic balance of electrostatic force and surface tension. This is represented by Rayleigh’s derived theory of instability of charged droplets. According to his theory, the equation for instability is

1 $${\mathrm{Q}}={(8{\pi }^{2}{\varepsilon }_{0}\gamma {d}^{3})}^{1/2},$$

where Q is the charge on the droplet, ε_0_ is the permittivity of the surrounding gas, γ is the surface tension of the liquid, and *d* is the droplet diameter. When the electrostatic force exceeds the surface tension of the liquid, the charged droplets emitted from the apex of the Taylor cone undergo Coulomb fission into fine droplets, thereby generating nano/micro-scale particles. The electrospraying process has been regarded as a useful technology for producing various nano/microparticles owing to the use of simple equipment and good material compatibility^4^. Furthermore, by altering the process parameters such as flow rate, applied voltage, and nozzle tip, the electrospraying process can generate micro/nanoparticles of sizes ranging from sub-microns to millimeters^5^. For this reason, electrospraying is being actively studied, and the associated technology is being applied in various fields such as electronics^6^, biological systems^7,8^, solar cells^9,10^, food^11,12^, and energy conversion^13^.

Zeleny, a pioneer in electrospray research, discovered several modes of electrospray process for generating aerosols with different characteristics^14^. Many theoretical and experimental studies have since been conducted to examine different modes of electrospray^15–17^. The electrospray mode was classified primarily into dripping mode and non-dropping mode^18^. In a broader sense, it can be categorized into dripping, pulsating, and cone-jet modes. The parameters that determine the modes of the electrospray process include not only processing parameters such as viscosity of the solution, electrical conductivity, flow rate, applied voltage, and geometry of the system, but also environmental factors including humidity, temperature, and solvent concentration in the air^19^. Given that external environmental factors significantly influence the mode of the electrospray, even with identical process parameters, the mode of the electrospray process may sometimes be inconsistent. Furthermore, the mode of the electrospray process is sensitive to various parameters and factors, and the estimation of the electrospray process mode is complex. As this can significantly affect the quality of the produced micro/nanoparticles, it is necessary to accurately estimate the electrospray mode.

There are several methods to observe the electrospraying mode. Zeleny was the first to describe a snapshot of the electrospray mode^20^. He presented spray patterns of alcohol and glycerin with photographs and hand drawn sketches. Subsequently, the dynamic motions of the electrospray process were captured via stroboscope technology. Given that the electrospray process is an instantaneous phenomenon, a low-frame-rate camera with stroboscopy can only provide snapshots of random events during the electrospray. With advances in imaging devices, high-speed cameras and expensive optical equipment have been increasingly used to observe the time-resolved behavior of the electrospray process in high-time resolution^21^. However, the equipment for these observations is generally complex and expensive. Furthermore, as mode discrimination can generally only be ascertained by an experienced experimenter, real-time feedback on mode discrimination is not feasible. Therefore, for industrial applications, developing an efficient and real-time method for mode discrimination is necessary for the stable production of electrosprayed particles.

Convolution neural networks (CNNs) are representative, specialized, deep learning techniques for image feature extraction^22^. Several studies on image classification using CNN have been conducted, the majority of which have focused on discriminating between 2D image data^23–25^. Recently, in industrial and manufacturing engineering, CNN using 1D data, such as sound waves^26^ and vibration signals^27^ have been studied. Although CNN has produced outstanding results in discrimination, estimation, and prediction, its inherent complexity makes it difficult to identify discriminative cues^28^. One approach to specify discriminative cues, called the region of interest (ROI), uses a class activation map (CAM). CNN with CAM can identify the area on which the CNN model focuses in the form of a heat map, which facilitates determining the part related to the discrimination.

This study represents the first attempt at utilizing electric current to estimate the electrospray process mode. Based on electric current measurements, we propose a simple and automated mode-detection technique employing 1D CNN. The electrospray modes were classified into three types: dripping, pulsating, and cone jets. In this study, we used ethanol for the electrospray process, because ethanol is one of the representative solutions that can clearly exhibit various electrospray modes^29,30^. The solution flow rate and applied voltage were selected as experimental variables. The recorded current data are converted into frequency data through a Fourier transform, and then learned through a 1D CNN to determine the electrospray mode. The trained model was validated based on the evaluation metrics of accuracy, precision, recall, and F1 score. It was possible to check the black box of the 1D CNN for the mode determined through CAM, and it was found that there were characteristic parts for each mode. The proposed method can discriminate modes more accurately and precisely based on data compared to estimation based on human experience.

## Experimental sections

### Electrospray setup

Ethanol solution (≥ 99.9%, Daejung Chemicals, Korea) was filled in a 10 ml syringe (HENKE-JECT, LCK Tech Co., Korea) and ejected through a 23-gauge metal needle at a constant flow rate using a syringe pump (EP100, NanoNC, Korea)^31^. An electroconductive collector of aluminum tape on a poly (methyl methacrylate) (PMMA, Acryl Choika, Korea) substrate with dimensions (width × length) of 50 × 50 mm^2^ was prepared. A high voltage was applied between the metal needle and collector using a power supply (HV30, NanoNC, Korea), and the distance between the metal needle and collector was 190 mm. The electrospray process was conducted at a temperature of 20–25 °C and relative humidity of 40–50%^32^. In addition, a charge-coupled device (CCD) camera was mounted at the height of the nozzle tip to record a video of droplet ejection.

### Data collection and preprocessing

**Figure 1:**
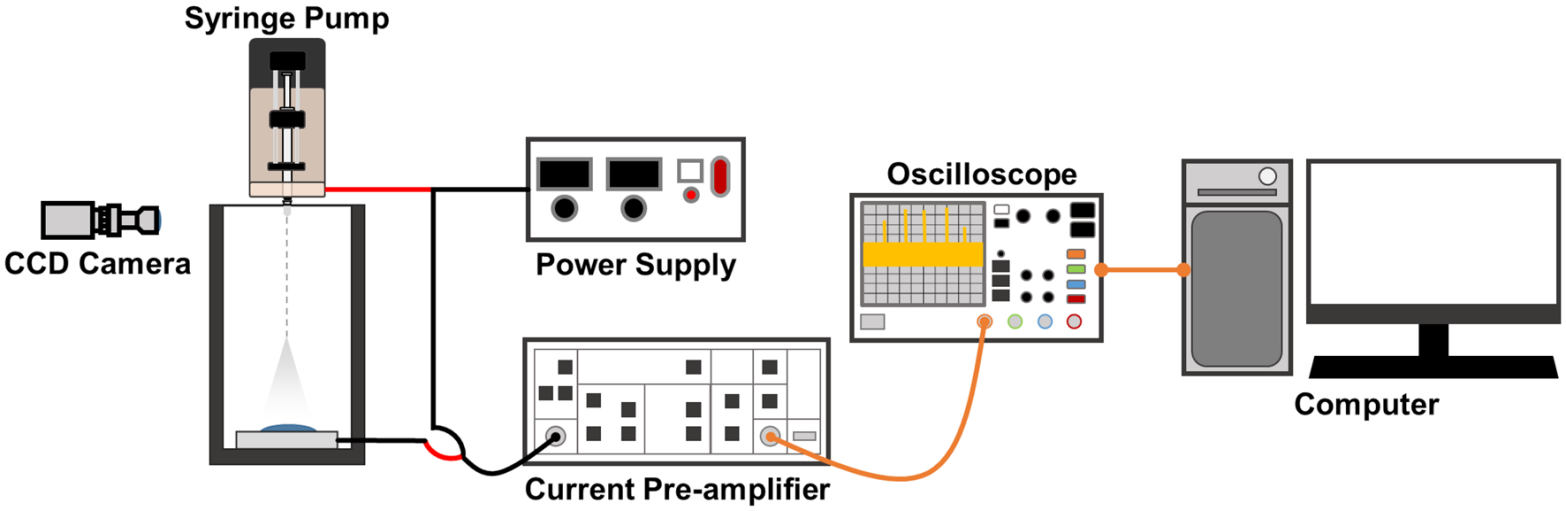
Schematic of experimental setup for current measurement.

**Figure 2:**
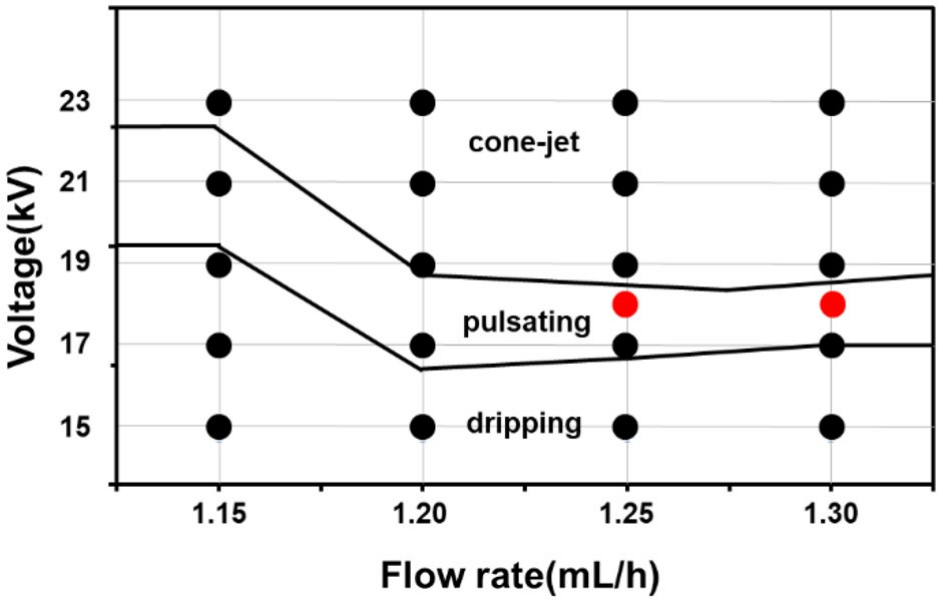
Experimental conditions in terms of voltage and flow rate.

The experimental setup is illustrated in Fig. 1. The current signal was measured by changing the flow rate from 1.15 to 1.30 ml/h in an increment of 0.05 ml/h, and the applied voltage from 15 to 23 kV in an increment of 2 kV. A low-noise current preamplifier (SR570, Stanford Research Systems, U.S.A.) was connected to the ground line of the power supply, which in turn was connected to the collector to measure the current signal. Subsequently, the measured current signal was converted into a voltage signal, which was measured using an oscilloscope (DSOX1204G, Keysight, U.S.A.). The current signal was measured at a sampling frequency of 2 kHz for 25 s. To check the periodic characteristics of the stored data, a fast Fourier transform (FFT) was performed using the SciPy fftpack module in Python and converted into frequency data. Figure 2 shows the experimental conditions with changing the flow rate and voltage to obtain datasets for three modes of the electrospray process: cone-jet, pulsating, and dripping. The black dots indicate the experimental conditions, and the boundaries divided the regions for each mode. The red dots indicate the additional experimental conditions for the pulsating mode. For each experimental condition, at least 9 experimental data were collected. Table 1 shows the composition of the entire datasets used in the 1D CNN with CAM. The dataset of each mode was divided into training and test datasets at a ratio of about 3:1. The dimension of the input data, which are the amplitude values of the frequency ranging from 0 to 40 Hz, was 641.

**Table 1:** The number of datasets for electrospray mode prediction.

Mode class	# of dataset	# of train dataset	# of test dataset
Dripping	92	72	20
Pulsating	40	31	10
Cone-jet	84	60	24
Total	217	163	54

### Model design of 1D CNN with CAM and prediction of electrospray mode

**Figure 3:**
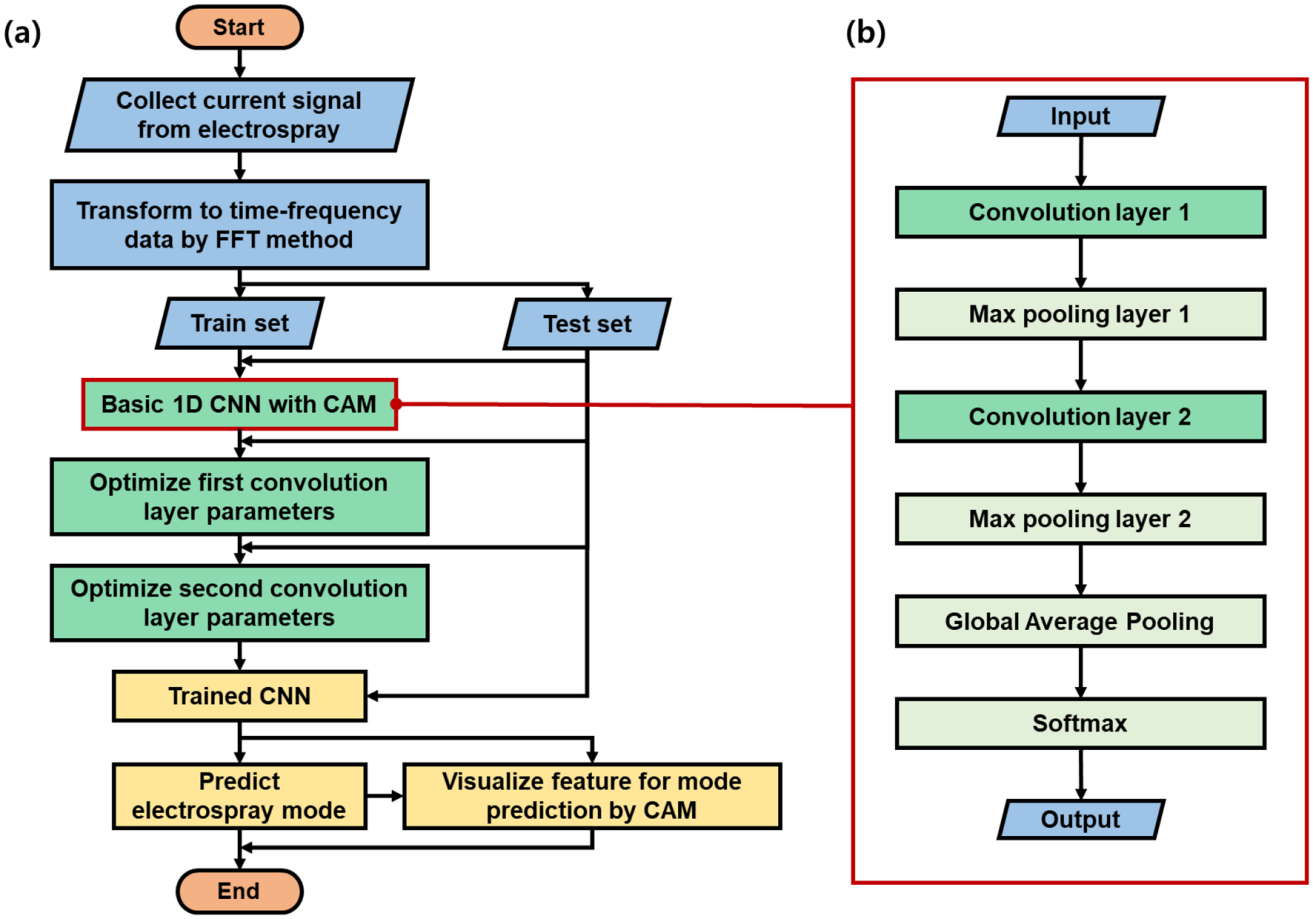
(**a**) Preparation of 1D CNN with CAM for electrospray mode prediction. (**b**) Detailed structure of 1D CNN with CAM.

The conventional CNN configuration was divided into two main stages, namely feature extraction and classification. Raw input data were fed into the convolution layers to extract features^22^. Pooling layers are frequently introduced between convolution layers to downsize convoluted features. Next, the extracted features were passed to the fully connected (FC) layers to classify the raw input data into a certain class. To pass the extracted features from the convolution layer to the FC layers, the extracted features with multiple dimensions or channels must be flattened into 1D data, which results in a loss of the location information of the raw input data. Such sequential convolution and FC layers of the CNN model render the raw input data more abstract^33^. To address these problems, the network structure of CAM was proposed to identify discriminative areas for the prediction of class^28^. For CAM, classification is performed by applying global average pooling (GAP) to each channel, instead of the flattening process^34^. This network structure can visualize the discriminative area in raw input data by multiplying the weight of the extracted feature map. The process is formulated as follows:

2 $${M}_{c}\left(x,y\right)=\sum_{k}{\omega }_{k}^{c}{f}_{k}(x,y),$$

where ${\omega }_{k}^{n}$ is the weight of class *c*, *k* is the number of channels, *f*_*k*_(*x, y*) is the value of the extracted feature value located at (*x, y*) of the *k*-th channel, and *M*_*c*_(*x, y*) is the CAM for class *c*. Specifically, *M*_*c*_ represents the importance of the extracted feature map for classification into class *c.* Figure 3 depicts the steps in model preparation and training. First, the frequency data was prepared by converting the current signal collected during electrospray process. After that, a train set of the frequency data was used to train the 1D CNN with CAM. The 1D CNN with CAM is depicted in Fig. 3b. The frequency data passed through two convolution layers and the GAP layer of the 1D CNN with CAM, sequentially, and was utilized for the electrospray mode prediction via the softmax operation. Next, the parameters of the first and second convolution layers of the 1D CNN with CAM were sequentially optimized by changing the number of channels gradually doubling from 8 to 128. A 1D CNN with a single convolution layer was utilized to find the optimal number of channels for the first convolution layer. We altered the number of channels of the 1D CNN with a single convolution layer and selected the number of channels exhibiting the highest accuracy. Subsequently, the 1D CNN with two convolution layers was optimized by fixing the first convolutional layer and finding the optimal number of the channels of the second convolution layer. Finally, with the optimized 1D CNN with CAM, the electrospray mode was predicted at high accuracy, and the discriminative cue of the mode prediction was presented based on the CAM heatmap. Training was initialized with a learning rate of 0.001, the optimization function was applied using TensorFlow Adam, and the learning model was trained for 2000 epochs. The computer, which was utilized to train the 1D CNN model with CAM, was configured based on Intel® Core™ i5-10400 CPU @ 2.90 GHz. The 1D CNN with CAM algorithm was realized with Python (version 3.8.7) and TensorFlow 2.0 under the Visual Studio Code framework.

## Result and discussion

**Figure 4:**
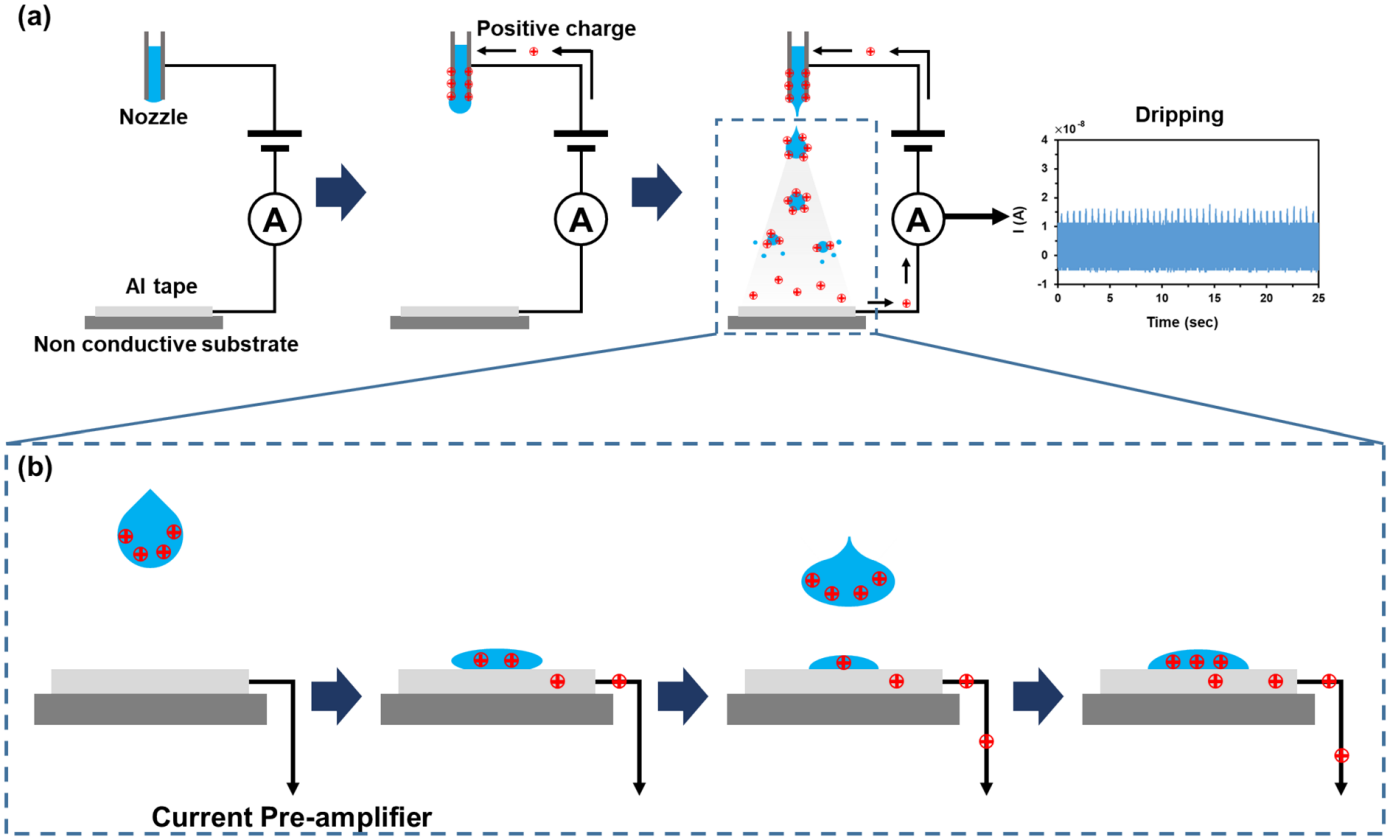
(**a**) Schematic of electrospray process. (**b**) Schematic showing the process of transfer of positive charges in ethanol.

Figure 4a shows a schematic of the experimental setup used for the electrospray process. Applying a high voltage between the nozzle and conductive collector of the Al tape results in the electrospray process, thereby ejecting highly charged droplets from the nozzle. Subsequently, the highly charged droplets were attracted to the conductive collector owing to the electrostatic force. When the droplets touched the conductive collector, the charge was transported from the highly charged droplet to the conductive collector, generating a charge flow, that is, an electric current. Furthermore, the remaining charges of the droplet would attract opposite charges toward the conductive collector to satisfy electro-neutrality, which also generates electric currents. The electric current at the electroconductive collector was measured as shown in Fig. 4b.

**Figure 5:**
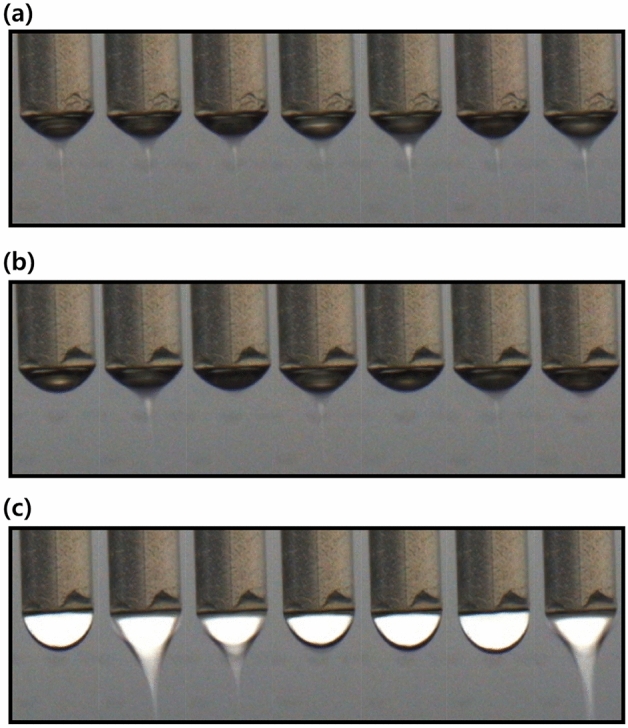
CCD camera snapshots of electrospray mode: (**a**) cone-jet, (**b**) pulsating, (**c**) dripping.

**Figure 6:**
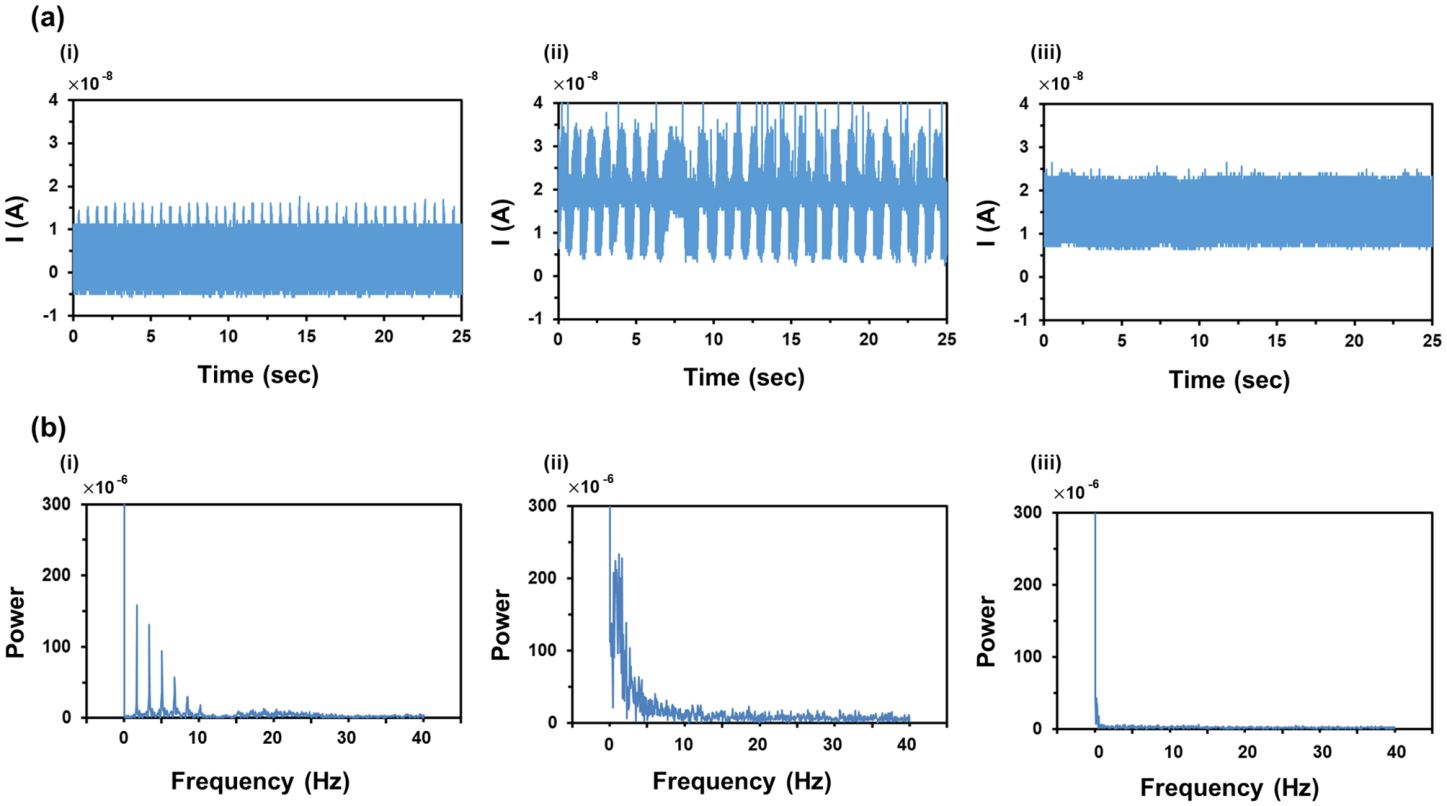
Measured signals of (**a**) current data and (**b**) frequency data. (**i**) dripping, (**ii**) pulsating, and (**iii**) cone-jet.

Figure 5 presents the three modes of the electrospinning process: cone-jet, pulsating, and dripping. The dynamic behaviors of the cone-jet, pulsating mode, and dripping mode were captured with a CCD camera during the electrospray process, as shown in Fig. 5a–c, respectively. Figure 6a and b show the measured current data and converted frequency data of the dripping, pulsating, and cone-jet modes. From the current data, we can observe periodic peaks in the dripping and pulsating modes. The amplitude of the peak of the dripping mode was slightly less than that of the pulsating mode. Generally, the dripping mode was observed at a lower applied voltage; thus, the generated droplet in the dripping mode would possess a lesser charge compared to that of the pulsating mode. In the case of the dripping mode, a harmonic structure was observed in the frequency data because the droplets periodically plunged. By contrast, in the case of the pulsating mode, current peaks appeared periodically, but the frequency appeared to be slightly irregular. The irregular behavior of the current data was confirmed by the frequency data, which exhibited numerous peaks between 0 and 5 Hz owing to irregular trends. Finally, in the case of the cone-jet, the overall electric current was shifted upward, given that the cone-jet mode was observed at a higher electric voltage than the other modes. Because the cone-jet mode produced a number of extremely small droplets compared to the other modes, it was difficult to recognize any distinguishable peaks. In this regard, we selected the frequency range of 0–40 Hz because the major peaks of dripping and pulsating modes were located within that range. Considering that the cone-jet mode can be divided into more specific modes of tilted-jet, twin-jet, and multi-jet, the utilization of a wider range of frequency data would facilitate discrimination of such diverse electrospray modes. As this study represents the first attempt at mode discrimination using electric current and CNN, we focused on the discrimination among the three modes of the electrospray process. The collected frequency data exhibited slight data imbalance originated from the narrow processing window of the pulsating mode. To alleviate such data imbalance, additional experiments, which are denoted as red dots at Fig. 2, were performed to increase the number of the frequency data of the pulsating mode.

**Figure 7:**
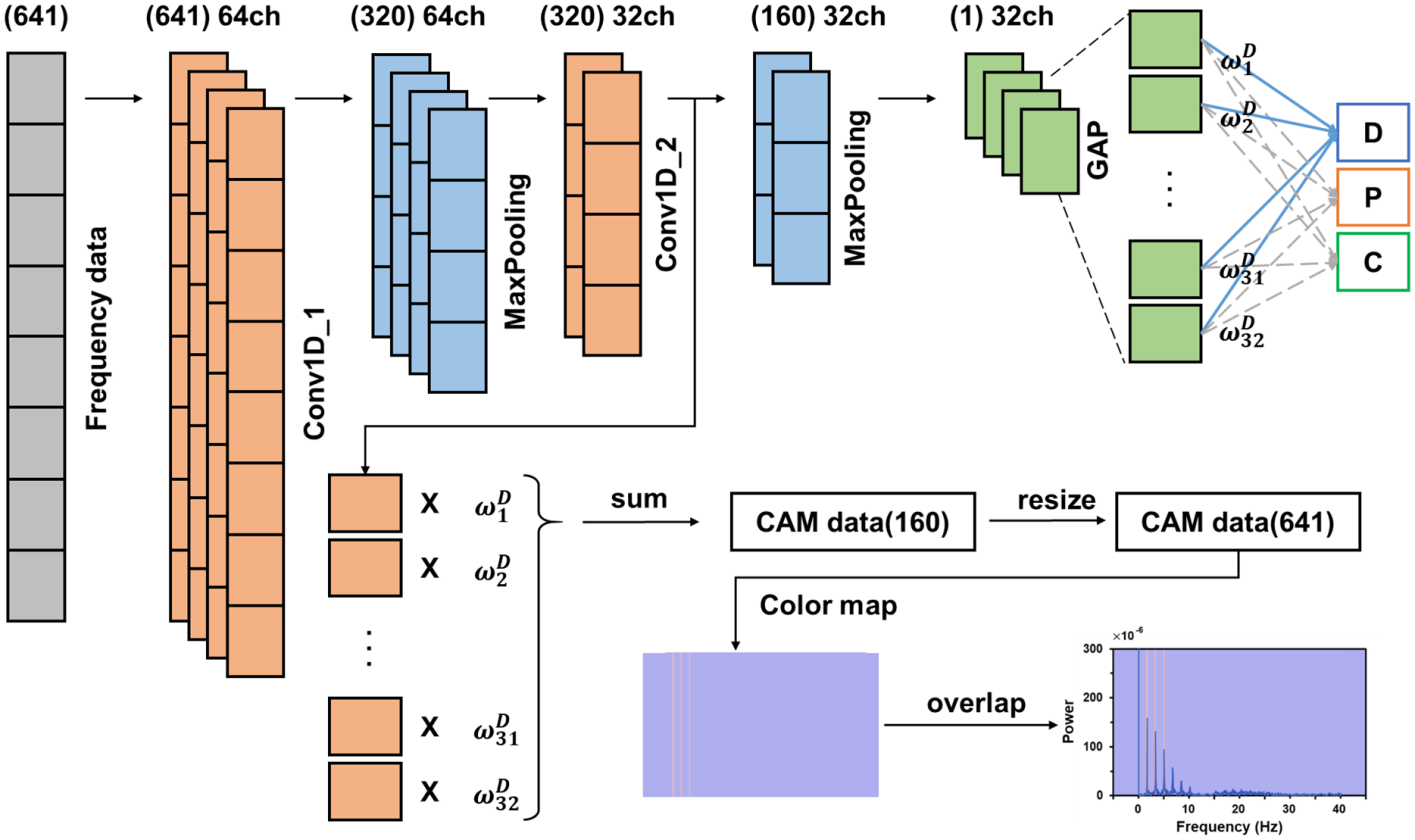
Architecture of CNN with CAM for electrospray mode prediction.

Figure 7 shows the detailed network structure of the CNN with CAM. The network structure of a CNN with CAM consists of two convolutional layers, two pooling layers, and a GAP layer. A rectified linear unit (ReLU) was used as the activation function of the convolution layer, and batch normalization was performed to prevent overfitting. In addition, zero padding was performed to maintain the dimensionality of the output value and a pooling layer was added to improve the discrimination performance after each convolution layer. Because the output represents the prediction of the electrospray mode, it is categorized into three classes: dripping, pulsating, and cone-jet modes. The output was in the form of a list of probabilities for each mode, and the mode corresponding to the largest value in the list was used as the prediction mode. CAM data are created by multiplying the feature map that has undergone the second convolution layer by the weight that determines the corresponding mode, and then adding and resizing it to fit the size of raw input data. After converting the created data into a heat map, it was overlapped on the graph of corresponding raw input data.

The optimization process was performed by changing the number of channels that greatly affect the feature abstraction and extraction among various parameters of the convolution layer. Table 2 shows the performance of the 1D CNN with a single convolution layer according to the number of channels. Given that a single convolution layer with 64 channels exhibited the best performance, the number of the channels of the first convolution layer was set to 64. Next, the 1D CNN with two convolution layers exhibited the superior performance when the number of channels in the second convolution layer was 32 with the optimized first convolution layer (Table 3). In this regard, the 1D CNN with CAM was tuned with the optimal first and second convolution layers with the numbers of channels of 64-32. The detailed network structure and model parameters are summarized in Table 4.

**Table 2:** Performance of 1D CNN with a single convolution layer according to different number of channels from 8 to 128.

Architecture	Test accuracy (%)	Test precision (%)	Test recall (%)	Test F1-score (%)
Conv1D (8)	62.69	70.78	63.33	64.44
Conv1D (16)	72.22	72.13	66.39	67.51
Conv1D (32)	74.07	69.84	70.56	69.44
Conv1D (64)	75.93	71.62	71.94	70.82
Conv1D (128)	74.07	67.44	67.22	67.30

**Table 3:** Performance of 1D CNN with two convolution layers according to varying number of channels of the second convolution layer from 8 to 128 with the optimized first convolution layer with 64 channels.

Architecture	Test accuracy (%)	Test precision (%)	Test recall (%)	Test F1-score (%)
Conv1D(64) + Conv1D(8)	94.44	96.30	90	92.16
Conv1D(64) + Conv1D(16)	96.30	97.08	93.33	94.80
Conv1D(64) + Conv1D(32)	96.30	97.44	93.33	94.96
Conv1D(64) + Conv1D(64)	94.44	96.30	91.67	93.48
Conv1D(64) + Conv1D(128)	94.44	95.75	91.94	93.44

**Table 4:** Parameters of the proposed CNN model for electrospray mode prediction.

Layer	Type	Channels	Data size of each channel	Kernel size	Stride
0	Input	1	641	–	–
1	1D convolution	64	320	3	1
2	1D maxpooling	–	320	2	2
3	1D convolution	32	160	3	1
4	1D maxpooling	–	160	2	2
5	Global average pooling	32	–	–	–
6	Linear	3	–	–	–

**Figure 8:**
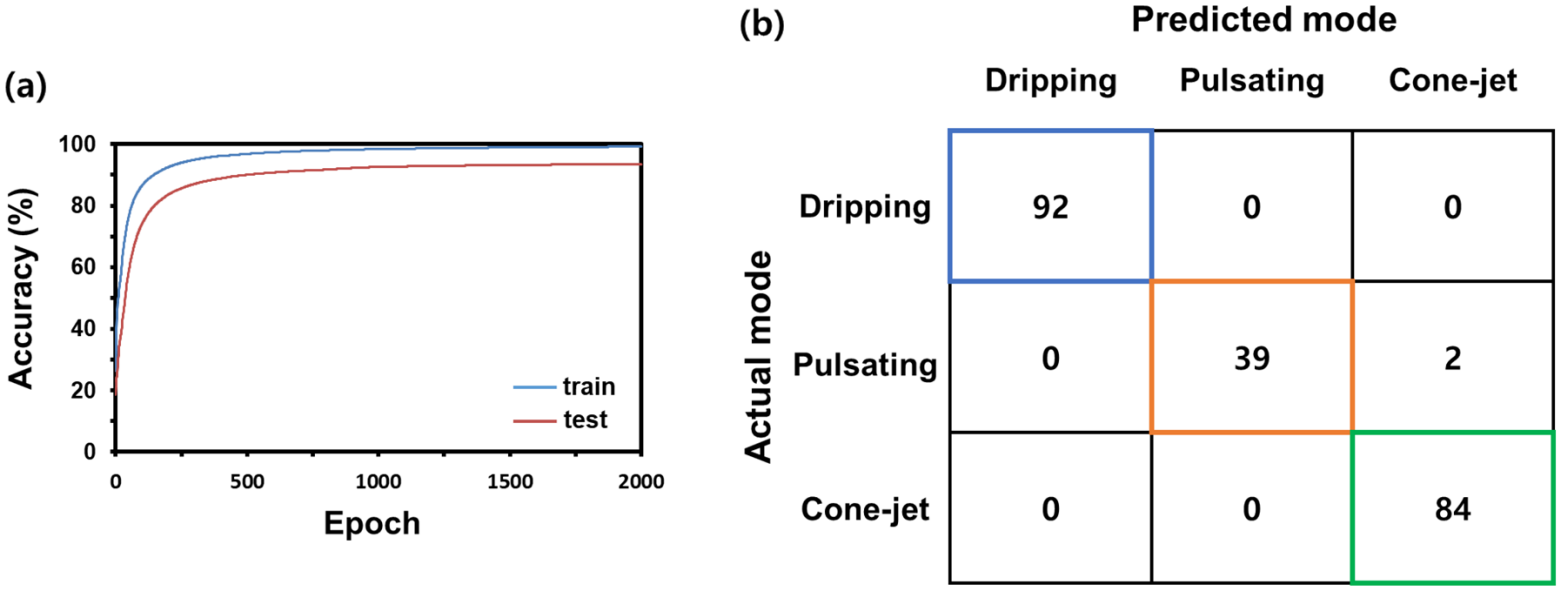
(**a**) Training result of 1D CNN with CAM and (**b**) confusion matrix for all datasets.

Figure 8a shows the train and test accuracy according to epochs of the 1D CNN with CAM. It shows that both train and test accuracy was converging to a sufficient level as the training progressed. Given that the train and test accuracy seemed to be saturated after 2000 epochs, we ended the training at 2000 epochs to obtain high accuracy and prevent the possibility of over-fitting. Figure 8b shows the confusion matrix of the trained 1D CNN with CAM with both train and test datasets. All cases except for two cases in the test dataset was correctly predicted by the trained 1D CNN with CAM. We found that even with a small number of epochs (2000), the 1D CNN with CAM exhibited high accuracy in the mode prediction. We presumed that such a small number of epochs for high accuracy would be attributed to the small size of the datasets and obvious discriminative cues.

Table 5 showed the effectiveness of our proposed deep learning model, compared to other shallow machine learning algorithms, i.e., kernel support vector machine (SVM), multilayer perceptron (MLP), random forest (RF) and logistic regression (LR). The learning parameters of each machine learning algorithm are shown in Table S1. The 1D CNN with CAM yielded an exceptional result at the highest performance of 96.30% accuracy, 97.44% precision, and 93.33% recall and 94.96% F1-score without experts’ prior knowledge and manual design of the features.

**Table 5:** Performance of several classifiers for electrospray mode prediction.

Classifier	Accuracy (%)	Precision (%)	Recall (%)	F1-score (%)
1D CNN with CAM	96.30	97.44	93.33	94.96
Kernel SVM	94.44	96.30	90	92.16
Multilayer perceptron	85.19	88.65	82.78	84.69
Random forest	90.74	90.48	85	86.78
Logistic regression	90.74	90.48	85	86.78

**Figure 9:**
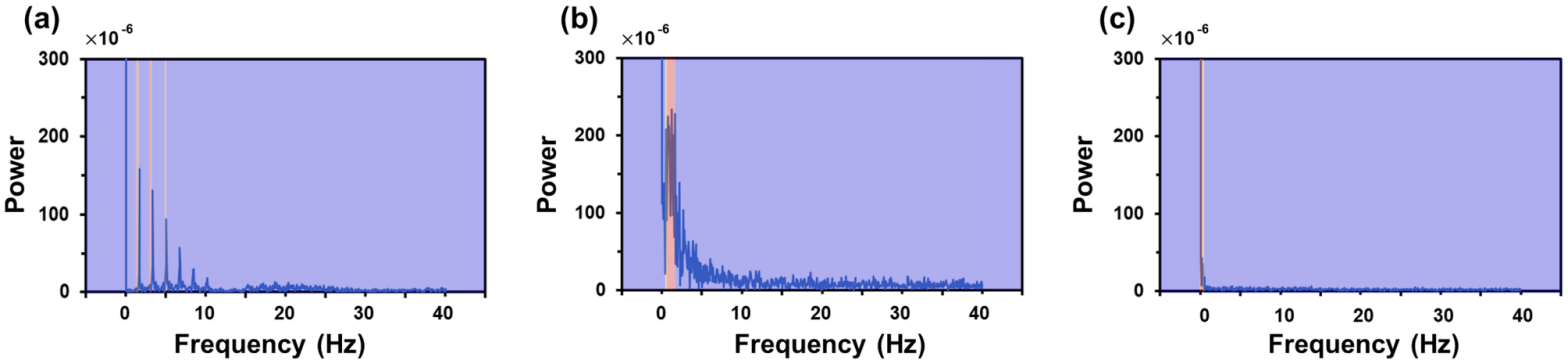
Images of overlapping CAM heat map on frequency data: (**a**) dripping, (**b**) pulsating, and (**c**) cone-jet.

Figure 9 shows frequency data overlapped with the CAM heatmap for each mode. Figure 9a–c show the dripping, pulsating, and cone-jet modes, respectively. As the CNN is trained, the part with high concentration is shown in red, and the part with low concentration is shown in blue. In the case of dripping mode, the trained model focused on frequencies of the harmonic structure, except for the 0 Hz data, and in the case of the pulsating mode, the interest in frequency range of 0–5 Hz was high. In addition, it was found that, in the case of cone-jet mode, a lot of attention was paid to the 0 Hz value, which indicates shifting of current data due to high voltage. Evidently, the learning model is similar to judging based on physical characteristics rather than evaluating a mode in the form of a black box; moreover, it exhibits excellent performance.

## Conclusion

We present a simple and automated electrospray mode detection method based on a 1D CNN and elucidate the rationale for mode discrimination using CAM. During the electrospray process, the mode was determined only based on current data generated by the electrospray, without directly observing the shape of the jet with the naked eye or optical equipment. After converting the collected current data for each mode into frequency data using FFT, it was confirmed that each mode has a characteristic frequency. Through trial and error, we found an appropriate learning model that was simple and effective for mode discrimination. In the existing CNN model, the cause of the learning result could not be determined because the learning process was a black box, but the basis for mode discrimination was confirmed using CNN with CAM. CAM also showed that the mode was discriminated based on the characteristic frequency of each mode. If the mode detection method presented in this paper is used, the mode can be identified in real time without individually checking the nozzle tips during the electrospray process.

## Supplementary Information

Supplementary Table S1.

## Data Availability

The datasets used and/or analysed during the current study available from the corresponding author on reasonable request.
