# Supplementary material for: Electrospray mode discrimination with current signal using deep convolutional neural network and class activation map
Source: Sci Rep. 2022 Sep 29;12:16281. doi: 10.1038/s41598-022-20352-y (PMC9523038; doi:10.1038/s41598-022-20352-y)
Supplement: Supplementary file 1 — Supplementary Table S1. [file 41598_2022_20352_MOESM1_ESM.docx]

Supplementary Information for

**Electrospray Mode Discrimination with Current Signal using Deep Convolutional Neural Network and Class Activation Map**

Man Jin Kim^1^, Jin Yeong Song^1^, Seok Hyeon Hwang^1^, Dong Yong Park^2^, Sang Min Park^1,^*

^1^School of Mechanical Engineering, Pusan National University, 2, Busandaehak-ro 63 beon-gil, Geumjeong-gu, Busan, 46241, Republic of Korea

^2^Smart Manufacturing Technology R&D Group, Korea Institute of Industrial Technology, 320 Techno sunhwan-ro, Yuga-eup, Dalsung-gun, Republic of Korea

*corresponding author : [sangmin.park@pusan.ac.kr](mailto:sangmin.park@pusan.ac.kr)

Keywords: Electrospray; Spray mode; 1D CNN; CAM

| **Kernel SVM** | **Multilayer perceptron** |
| --- | --- |
| Kernel : rbf | # of hidden nodes : 200 |
| Max_iteration : no limit | Activation : relu |
| *C* : 50 | Solver : adam |
| Tolerance : 0.001 | Max_iterations : 2000 |
| **Random forest** | **Logistic regression** |
| Max_features : sqrt | Penalty : 12 |
| Min_samples_leaf : 1 | Solver : newton-cg |
| # of tree : 1000 | C : 0.01 |
| Min_samples_split : 2 | Max_iterations : 2000 |

**Table S1.** Training parameters of the four machine learning algorithms
